# Protein Supplementation Does Not Maximize Adaptations to Low-Volume High-Intensity Interval Training in Sedentary, Healthy Adults: A Placebo-Controlled Double-Blind Randomized Study

**DOI:** 10.3390/nu14193883

**Published:** 2022-09-20

**Authors:** Dejan Reljic, Nilas Zieseniss, Hans J. Herrmann, Markus F. Neurath, Yurdagül Zopf

**Affiliations:** 1 Department of Medicine 1, University Hospital Erlangen, Friedrich-Alexander-Universität Erlangen-Nürnberg, Ulmenweg 18, 91054 Erlangen, Germany; 2 Hector-Center for Nutrition, Exercise and Sports, Department of Medicine 1, University Hospital Erlangen, Friedrich-Alexander University Erlangen-Nürnberg, Ulmenweg 18, 91054 Erlangen, Germany; 3 German Center Immunotherapy (DZI), University Hospital Erlangen, Friedrich-Alexander University Erlangen-Nürnberg, Ulmenweg 18, 91054 Erlangen, Germany

**Keywords:** HIIT, cardiometabolic health, whey protein, cardiorespiratory fitness

## Abstract

There is ample evidence that specific nutritional strategies can enhance adaptions to resistance and endurance training. However, it is still unclear whether post-session protein supplementation may increase the effects of low-volume high-intensity interval training (LOW-HIIT). We examined the impact of LOW-HIIT combined with protein vs. placebo supplementation on cardiometabolic health indices in sedentary healthy individuals. Forty-seven participants (31.1 ± 8.0 yrs) performed cycle ergometer LOW-HIIT (5–10x1 min at 80–95% maximum heart rate) for eight weeks and randomly received double-blinded 40 g of whey protein (PRO-HIIT, *N* = 24) or an isocaloric placebo (maltodextrin, PLA-HIIT, *N* = 23) after each session. The maximum oxygen uptake (VO_2max_, primary outcome) and several secondary cardiometabolic outcomes were determined pre-/post-intervention. VO_2max_ increased in PRO-HIIT (+2.8 mL/kg/min, *p* = 0.003) and PLA-HIIT (+3.5 mL/kg/min, *p* < 0.001). Systolic and diastolic blood pressure decreased in PRO-HIIT (−7/3 mmHg, *p* < 0.05) and PLA-HIIT (−8/5 mmHg, *p* < 0.001). Gamma glutamyl transferase (−2 U/L, *p* = 0.003) decreased in PRO-HIIT and alanine aminotransferase (−3 U/L, *p* = 0.014) in PLA-HIIT. There were no significant between-group differences in any of the outcome changes. In conclusion, LOW-HIIT improved VO_2max_ and other cardiometabolic markers irrespective of the supplementation condition. Post-session protein supplementation does not seem to provide any additional benefit to LOW-HIIT in improving cardiometabolic health in sedentary healthy individuals.

## 1. Introduction

It is well established that regular physical exercise is of enormous value for the prevention and treatment of numerous chronic diseases. A physically inactive lifestyle is considered a major behavioral risk factor associated with a variety of health conditions [1,2,3,4]. In this context, low cardiorespiratory fitness (CRF, typically quantified as maximal oxygen uptake, VO_2max_) has been documented to be a major risk marker of cardiovascular disease development and all-cause mortality, much stronger than other cardiometabolic risk factors, such as increased body mass index (BMI), smoking, hypertension, or elevated cholesterol levels [5,6,7]. However, despite the overwhelming evidence for the beneficial health effects of being physically active, a large proportion of the adult population worldwide does not meet the recommended physical activity amounts (i.e., a minimum of 150 min of moderate or 75 min of vigorous-intensity aerobic physical activity per week [8]) [9] and this trend has been further amplified by the outbreak of the COVID-19 pandemic [10,11]. The individual reasons for physical inactivity are certainly diverse, but large-scale surveys conducted during the last decade on different continents have frequently revealed that “lack of time” constitutes a major barrier for regular physical activity and exercise [12,13,14]. Thus, in recent years, the development and evaluation of more time-efficient exercise modalities has increasingly become a focus in the research field of exercise physiology and sports science [15]. In this light, low-volume high-intensity interval training (LOW-HIIT) has emerged as a promising exercise type that has shown great potential in improving a variety of health outcomes with less time effort compared to more traditional exercise methods, such as (moderate-intensity) longer-distance endurance training [16,17,18]. LOW-HIIT is typically characterized by short, vigorous exercise bouts separated by low-intensity recovery periods and involves, per the previous definition, a maximal total session duration of ≤30 min (including warm-up, recovery periods, and cool-down) [17]. Previous research from our group has shown, for example, that LOW-HIIT can significantly improve VO_2max_ and various cardiometabolic markers in sedentary but otherwise healthy individuals [19] and in obese metabolic syndrome patients [20,21,22,23] after only a few weeks.

However, in contrast to longer-distance endurance training or resistance exercise [24], the potential of certain nutritional strategies and/or specific supplements to enhance the physiological adaptations in response to LOW-HIIT is still relatively sparsely investigated. It has been reported, for example, that there is evidence to suggest that several supplements, including sodium bicarbonate, nitrates, beta-alanine, caffeine and creatine, may provide potential benefits for improving HIIT adaptations [16,25]. Although it is generally accepted that athletes performing intense training, but also individuals engaged in recreational sports or general fitness routines, may require higher protein intakes compared to the sedentary population [24], there is still a paucity of data regarding the impact of targeted post-session protein supplementation on HIIT-induced exercise adaptations [25]. Previous research on the significance of proper protein intake is mainly based on studies using resistance training regimes, but it has also been documented that the consumption of a high-quality protein in doses of 20–40 g after a session of higher-volume endurance training may maximize muscle protein synthesis and support recovery processes [24]. The physiological adaptations occurring after endurance exercise include the formation of new capillaries, mitochondrial proteins and other proteins involved in oxygen transport (e.g., hemoglobin and myoglobin) [26,27]. These adaptations play a critical role in the development of CRF (in addition to cardiac and pulmonary adaptations) and may translate into improved exercise performance over the longer term [28,29]. Thus, for these adaptation processes to run optimally, individuals participating in cardiovascular training modalities may hypothetically benefit from a post-exercise supplementation in the form of protein. However, given that most of the previous studies investigating the effects of protein supplements in conjunction with endurance training have typically used higher exercise volumes, there is currently still a lack of data on whether individuals engaged in LOW-HIIT may also benefit from a targeted post-session protein supplementation. Given the constantly high popularity of (LOW-)HIIT in recent years [30], it appears timely to address this research gap. 

Therefore, the present study aimed to investigate the impact of an eight-week LOW-HIIT program combined with a post-session intake of 40 g of whey protein in comparison to LOW-HIIT combined with an isocaloric placebo on CRF and cardiometabolic markers in a cohort of previously sedentary, healthy individuals. As HIIT has been shown to be a particularly effective training method to increase mitochondrial biogenesis and protein levels of glucose, lactate, and fatty-acid transporters in the skeletal muscle [31], we hypothesized that targeted protein supplementation after LOW-HIIT would have beneficial effects on exercise-induced improvements in CRF and cardiometabolic outcomes.

## 2. Materials and Methods

### 2.1. Study Design

**Figure 1:**
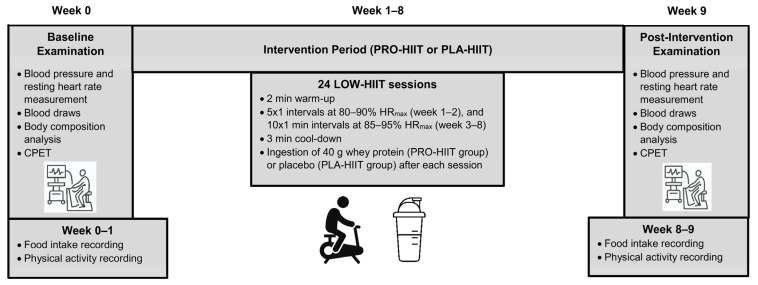
Study design overview.

This study was a placebo-controlled double-blind randomized trial over a period of 8 weeks. Participants were randomly allocated to either the LOW-HIIT plus protein supplementation group (PRO-HIIT) or the LOW-HIIT plus placebo supplementation group (PLA-HIIT). The primary outcome of the study was maximal oxygen uptake (VO_2max_). Secondary outcomes were several cardiometabolic markers and body composition variables as further specified in the Methods section. Outcome assessments were performed 1 week prior to the onset of the intervention (baseline examination, T-1) and 1 week after termination of the intervention (post-intervention examination, T-2). An overview of the study design is depicted in Figure 1. Group allocation was performed after the baseline examination using a computer-generated random number sequence (MinimPy, GNU GPL v3) by a researcher who was not involved in data collection. Prior to randomization, participants were stratified according to the primary outcome VO_2max_ (<35 mL/kg/min, and ≥35 mL/kg/min), age (<30 years, and ≥30 years) and gender (male/female) to achieve a more balanced distribution of participants’ main characteristics between both groups. Participants were fully informed about the aims and procedures of the study, which conformed to the Helsinki Declaration. Written consent was obtained from all participants before enrolment. The study protocol was approved by the Ethical Committee of the Medical Faculty of the Friedrich-Alexander University Erlangen-Nürnberg (approval number: 147_19B) and registered at ClinicalTrials.gov (ID-number: NCT04359342).

### 2.2. Participants

Participants were recruited through newspaper and internet advertisements. All interested persons were able to contact our research staff by mail or phone to discuss the criteria for study participation. If the criteria were formally met, then the study candidates were given an appointment for the baseline examination. In brief, inclusion criteria were as follows: age ≥ 18 years, a self-reported predominantly sedentary lifestyle as defined elsewhere [32], and no participation in any other exercise or nutrition intervention program. Exclusion criteria were: clinical diagnosis of heart disease, cancer, severe orthopedic conditions or other major health problems that might preclude safe participation in an exercise program, and pregnancy. All participants agreed to maintain their usual lifestyle patterns throughout the intervention period to minimize potential confounding effects. Based on our previous research [19], indicating a large effect (Cohen’s *d*  =  0.97) of LOW-HIIT on the primary outcome (VO_2max_) in previously untrained individuals, an a priori sample-size calculation revealed that 16 participants per group would be required to yield a power of 0.95 in a 2-sided test with a 5% level of significance (G*Power, Software version 3.1.9.7, Heinrich Heine University, Düsseldorf, Germany). To account for potential dropout, we aimed to recruit 25 participants per group.

### 2.3. Health Examinations

The baseline examination was conducted 1 week prior to the onset of the LOW-HIIT intervention. The post-intervention examination was carried out within the first week after termination of the 8-week LOW-HIIT period, at least 3 days apart from the last training session to ensure sufficient recovery and at a similar time of day as the baseline examination to avoid possible circadian effects. The examinations included blood pressure measurements, blood sampling, determination of body composition, and cardiopulmonary exercise testing (CPET). A 12-lead electrocardiography recorded at rest and during CPET was performed to ensure a safe participation in the LOW-HIIT program. All assessments were performed in a stable ambient environment under laboratory conditions at our research center and were strictly standardized as described below. Before each examination, participants were asked to arrive after having fasted overnight and to refrain from alcohol and vigorous physical activity for at least 24 h prior to the examinations. Care was taken that all female participants received their pre- and post-intervention examination during the same menstrual-cycle phase. All researchers who were involved in data collection were not aware of the participants’ group allocation.

#### 2.3.1. Blood Pressure and Resting Heart Rate Measurements

After arrival to the laboratory, participants were first asked to empty their bladder and thereupon, to rest for 5 min seated. Thereafter, participants’ systolic (SBP) and diastolic (DBP) blood pressure values were measured using a validated, automatic upper-arm BP device (M5 professional, Omron, Mannheim, Germany) [33]. As recommended by the American College of Cardiology/American Heart Association [34], two consecutive measurements were performed on both arms in intervals of 60 sec and the averaged values of the arm with the higher BP were used for further analysis. Additionally, mean arterial blood pressure (MAB) was calculated as follows:

MAB = DBP + (1/3[SBP − DBP])

#### 2.3.2. Blood Sampling

After BP measurements, participants remained in the seated position and blood samples were drawn via venipuncture from a vein in the antecubital fossa using a disposable cannula (S-Monovette, Sarstedt, Nürmbrecht, Germany). After collection, blood samples were immediately processed and transported to the diagnostic laboratories of the University Hospital Erlangen for further analyses. Blood samples were used to determine serum concentrations of fasting glucose, triglycerides, total cholesterol, low-density lipoprotein cholesterol (LDL-C), high-density lipoprotein cholesterol (HDL-C) and liver biochemistry (alanine aminotransferase (ALT), aspartate aminotransferase (AST) and gamma glutamyl transpeptidase (GGT)), which were measured photometrically (Clinical Chemistry Analyzer AU700 or AU5800, Beckman Coulter, Brea, CA, USA), and high-sensitivity C-reactive protein (hsCRP) and glycated hemoglobin A_1c_ (HbA_1c_) using turbidimetric immunoassays (Clinical Chemistry Analyzer AU700 or AU5800, Beckman Coulter, Brea, CA, USA, and COBAS Integra 400, Roche Diagnostics, Mannheim, Germany, respectively).

#### 2.3.3. Body Composition Measurements

A validated segmental multi-frequency bioelectrical impedance analysis (SMF-BIA) device (seca mBCA 515, Seca, Hamburg, Germany) [35] was used to determine participants’ body weight, body fat mass (BFM), body fat percentage (BF%), skeletal muscle mass (SMS) and total body water (TBW). Additionally, waist circumference (WC) was measured in an upright position to the nearest millimeter, at the approximate midpoint between the lower margin of the last palpable rib and the upper iliac crest along the midaxillary line, using a measuring tape.

#### 2.3.4. Cardiopulmonary Exercise Test (CPET)

The CPET was performed on a stationary electronically braked cycle ergometer (Corival cpet, Lode, Groningen, Netherlands) to determine VO_2max_, maximal power output (W_max_) and maximal heart rate (HR_max_). After a brief 1 min familiarization period, the test started at 50 W and afterwards, the work rate was gradually increased by 12.5 W per min for females and 15 W per min for males, respectively, until volitional exhaustion. Using this protocol, exhaustion was typically achieved within 8–12 in most participants, as recommended for CPET examinations [36]. During the CPET, heart rate (HR) was recorded continuously using a 12-lead ECG system (custo cardio 110, custo med, Ottobrunn, Germany). An open-circuit breath-by-breath spiroergometric system (Metalyzer 3B-R3, Cortex Biophysik, Leipzig, Germany) was used to measure oxygen uptake (VO_2_) and carbon dioxide output (VCO_2_). All VO_2_/VCO_2_ measurements were averaged over every 10 sec. Criteria to assume that maximal exhaustion was achieved by participants were the presence of at least two of the following: a plateau of VO_2_, a maximal respiratory exchange ratio (RER_max_) of ≥1.1, an age-predicted HR_max_ of ≥90% (using the equation: 220 − age) and a maximal rate of perceived exertion of ≥19 on the Borg scale [37], as suggested previously [38]. Subsequently, data acquired during the CPET were used to determine the individual target training HR zones for each participant, as specified below. Moreover, participants’ power output at the ventilatory threshold (W_VT_) was determined according to the V-Slope method (VCO_2_/VO_2_) [39] to assess submaximal exercise capacity.

### 2.4. Monitoring of Daily Nutrition and Physical Activity

Prior to study entry and during the last intervention week, participants were asked to log their daily nutritional intakes for three consecutive days using a standardized 24 h food record (Freiburger Ernährungsprotokoll; Nutri-Science, Freiburg, Germany). All dietary records were analyzed by a registered dietitian with the help of a computer-based software (PRODI 6 expert, Nutri-Science, Freiburg, Germany). Additionally, participants provided information about their daily physical activity behavior using an activity logbook. The respective metabolic equivalent (MET) intensity levels of each reported daily life physical activity were calculated and classified as light (<3 METs), moderate (3–6 METs), or vigorous (>6 METs), as suggested by Ainsworth et al. [40], and subsequently, the daily physical activity level (PAL) was estimated using the average MET score over 24 h. Based on the estimated PAL values and individual anthropometric data, participants were given individual nutritional recommendations to maintain a constant daily diet during the study period. The nutritional recommendations were based on the guidelines of the German Nutrition Society (DGE) [41,42]. In brief, resting metabolic expenditure (REE) was calculated according to the Harris and Benedict formula [43], as follows:

Men: REE (kcal/day) = 66.5 + 13.8 × weight (kg) + 5.0 × size (cm) − 6.8 × age (years)

Women: REE (kcal/day) = 655 + 9.6 × weight (kg) + 1.8 × size (cm) − 4.7 × age (years)

Total daily energy need was estimated by multiplying REE with PAL values. Participants were advised to consume 10–15% of their calories from protein, 30–35% from fat and ≥50% from carbohydrates [41]. To assists the participants in implementing the dietary recommendations at home, handouts with meal planning advice and detailed instructions were provided. 

### 2.5. LOW-HIIT Program

LOW-HIIT was performed 3x weekly on electronically braked cycle ergometers (Corival cpet, Lode, Groningen, The Netherlands) over a period of 8 weeks (a total of 24 sessions). The LOW-HIIT protocol was adapted from the protocol developed and previously described in detail by Reljic et al. [19]. In brief, participants initially warmed up at a low intensity for 2 min. The warm-up period was followed by 5 interval bouts at 80–90% HR_max_ during weeks 1–2, and then progressed to 10 interval bouts at 85–95% HR_max_ during weeks 3–8. Participants were instructed to increase the pedal cadence and/or the load resistance of the ergometer to reach their individually determined target HR during each interval bout. Each interval was divided by a 1 min recovery period of low intensity. Every session concluded with a 3 min cooldown phase at a self-paced low intensity (total time per session: 14 min during week 1–2, and 24 min during week 3–8, respectively). During each session, participants were equipped with a chest-strap HR monitor (acentas, Hörgertshausen, Germany) to track their HR responses in real time during exercise. HR values of every session were recorded and stored for later analysis by means of a specific HR monitoring and analysis system (HR monitoring team system, acentas, Hörgertshausen, Germany). All exercise sessions could be scheduled individually during the training center’s opening hours to maximize compliance, but participants were advised to rest one day between sessions to ensure proper recovery. All training sessions were supervised by certified sports- or physiotherapists who were previously trained in implementing the LOW-HIIT protocol.

### 2.6. Protein and Placebo Supplementation

Immediately after completion of each LOW-HIIT session, participants were administered in a double-blinded randomized manner either a supplement of 40 g whey protein (Fresubin Protein, Fresenius Kabi, Bad Homburg, Germany) in the PRO-HIIT group or an isocaloric, same-tasting maltodextrin placebo (MaltoCal 19, MetaX) in the PLA-HIIT group. The amount of protein supplementation was chosen because previous research has indicated that the ingestion of 40 g whey protein following exercise stimulates a significantly greater myofibrillar protein synthesis than 20 g in healthy individuals [44]. The caloric value and macronutrient composition of the supplements are shown in Table 1. Both supplements were mixed with low-fat milk (46 kcal/100 mL, 3.4 g protein, 4.8 g carbohydrates, 1.5 g fat) and served as a shake. One participant with lactose intolerance received the same milk in lactose-free form. Preparation and administration of the supplements was conducted by research staff not involved in data analysis. Participants recorded their individual consumption experiences with supplements, including taste rating and adverse events potentially associated with the ingestion (e.g., nausea, stomach pain). 

### 2.7. Statistical Analysis 

Statistical analyses of data were conducted using the software SPSS version 24.0 (SPSS Inc., Chicago, IL, USA). Initially, the normality distribution of data was examined with the Shapiro–Wilk test. Subsequently, a 2 × 2 repeated-measures ANOVA was performed to analyze the data for main effects of group (PRO-HIIT vs. PLA-HIIT), time (pre- vs. post-intervention), and interaction effects. Subgroup analyses were performed to check whether gender had any influence on changes in the primary outcome VO_2max_ and cardiometabolic markers. Levene’s test was used to verify homogeneity of variance. If significant main or interaction effects were found, then ANOVAs were followed by Holm–Sidak’s post-hoc tests for multiple comparisons and post-hoc paired *t*-tests to analyze between-group differences and within-group (pre-post) changes, respectively. If data were skewed, then a log or square-root transformation was carried out and the same above-described statistical tests were used with the transformed data. If data could not be normalized through transformation, then the Friedman two-way analysis of variance was performed, and, in case of significant results, followed by Dunn’s Bonferroni post-hoc tests for between-group comparisons and Wilcoxon’s and Mann–Whitney tests for within-group post-hoc comparisons. Additionally, effect sizes were calculated by means of the partial eta-squared (*ηp*^2^) for ANOVAs and Kendall’s coefficient of concordance (*W*) for the Friedman tests. Effect sizes were considered small (≤0.01), medium (≥0.06) and large ≥0.14 for *ηp*^2^, and small (≤0.10), medium (≥0.30), and large (≥0.50) for *W*, respectively, as previously suggested [45]. For all analyses, the significance level was set at *p* < 0.05. Data are displayed as means ± standard deviation (SD). Pre-/post-intervention changes are reported with 95% confidence intervals (95% CI).

## 3. Results

### 3.1. Study Flow, Compliance and Training Data

**Figure 2:**
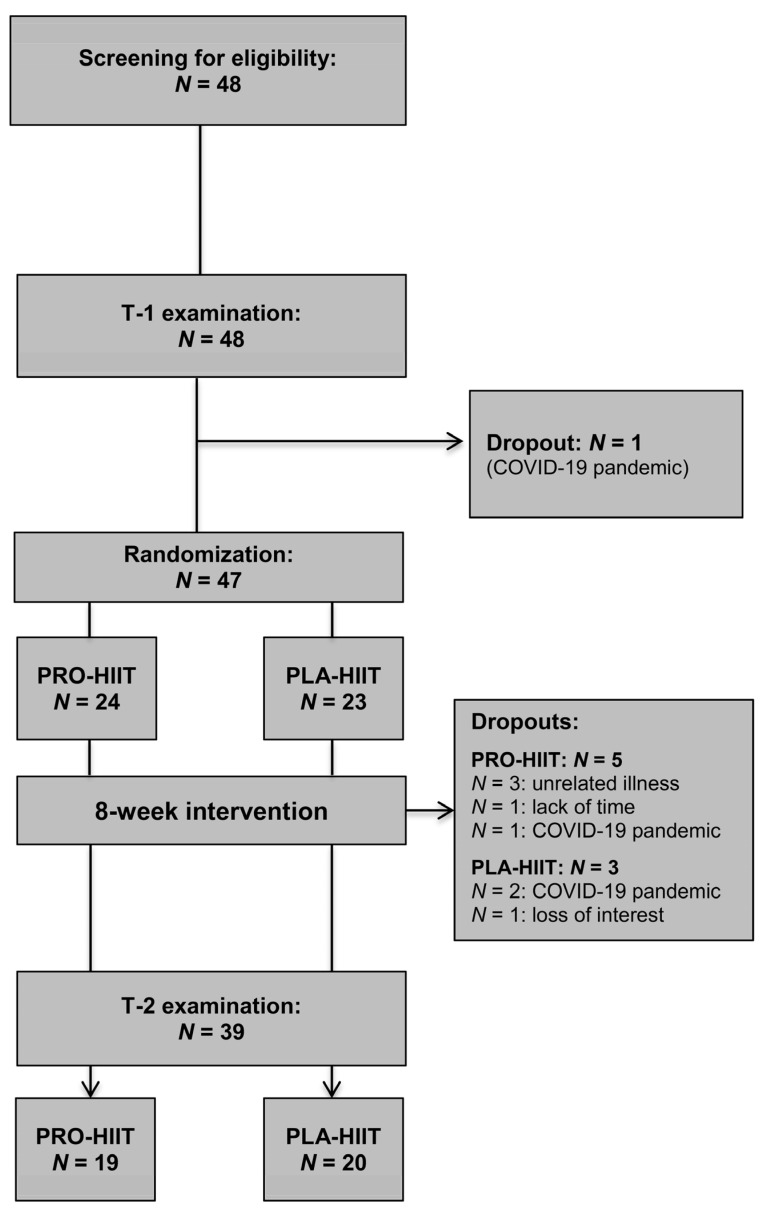
Study flow chart.

A total of N = 47 individuals (57% females) were included in this study (PRO-HIIT, N = 24, PLA-HIIT, N = 23). The baseline main characteristics were comparable between both groups (PRO-HIIT: 54% females, age: 30.0 ± 7.8 yrs, VO_2max_: 37.5 ± 5.6 mL/kg/min, and PLA-HIIT: 58% females, age: 32.5 ± 8.0 yrs, VO_2max_: 36.0 ± 7.1 mL/kg/min). All participants were free of medications, except for N = 3 women (PRO-HIIT, N = 1; PLA-HIIT, N = 2) taking contraceptive pills. During the intervention period, N = 8 participants dropped out (PRO-HIIT, N = 5, PLA-HIIT, N = 3). The specific dropout reasons are shown in Figure 2. Thus, the final analysis included a total of N = 39 participants (PRO-HIIT, N = 19, 47% females; PLA-HIIT, N = 20, 50% females). There were no significant baseline differences between the two groups. Moreover, no significant effects of gender on the changes in the primary outcome VO_2max_ or cardiometabolic markers were found, and therefore, the results of both males and females were considered together in all analyses. Training compliance (i.e., scheduled vs. attended sessions) was very high in both groups (PRO-HIIT: 92 ± 8%, PLA-HIIT = 92 ± 8%). The average peak HR achieved within the intervals corresponded to 95 ± 3% of HR_max_, verifying that the prescribed exercise intensity was successfully achieved by the participants. The average HR during the exercise sessions (including warm-up, intervals, recovery phases and cool-down) corresponded to 79 ± 4% of HR_max_. During the study, no adverse events occurred that were related to LOW-HIIT. The majority of participants stated that they enjoyed engaging in LOW-HIIT. On a 7-point rating scale (1 = not enjoyable at all, and 7 = extremely enjoyable) the exercise program was rated with an average score of 5.7 ± 1.0 and 88% of participants reported that they intend to continue with LOW-HIIT on their own after the termination of the study.

### 3.2. Daily Nutrition and Physical Activity

There were no significant within- or between-group differences in daily nutrition and physical activity levels. The group-specific nutritional intake and physical activity values that were recorded pre-intervention and during the last week of the intervention are shown in Table 2.

### 3.3. Body Composition Data

No significant within- or between-group difference were detected for any body composition parameter. Group-specific pre-/post-intervention body composition data are shown in Table 3. 

### 3.4. Cardiorespiratory Fitness and Blood Pressure Measures

**Figure 3:**
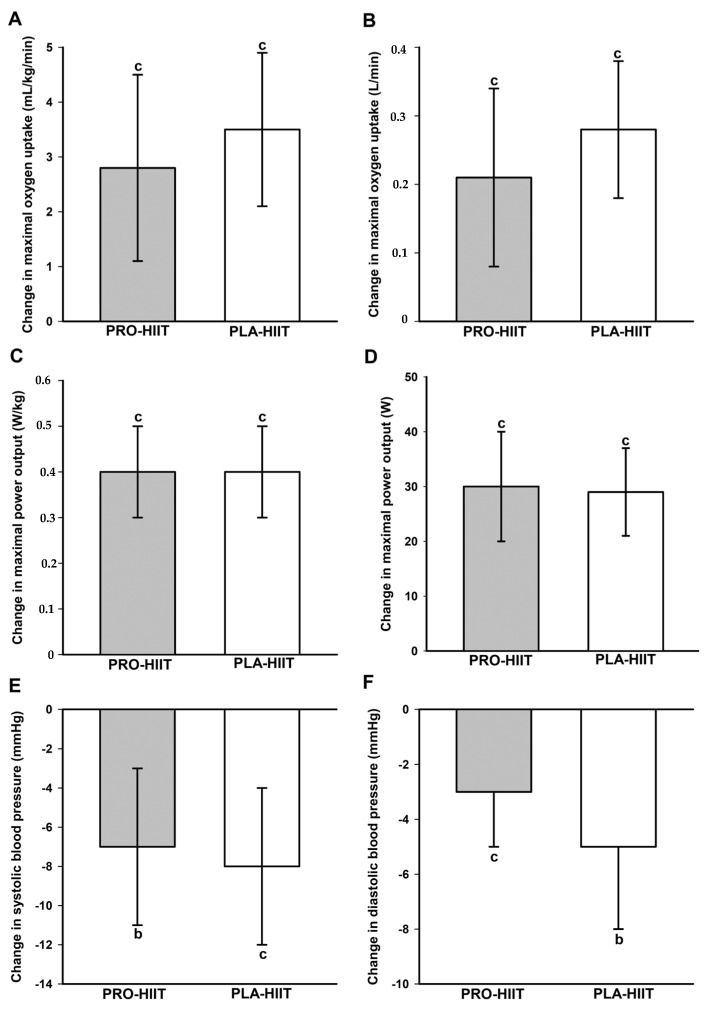
Changes in relative (**A**) and absolute (**B**) maximal oxygen uptake, relative (**C**) and absolute (**D**) maximal power output, and systolic (**E**) and diastolic (**F**) blood pressure. ^b^ (*p* < 0.01), ^c^ (*p* < 0.001): significant change between pre- and post-intervention. No significant between-group differences.

A significant main effect of time was found on relative (*p* < 0.001, *ή*^2^ = 0.50) and absolute VO_2max_ (*p* < 0.001, *ή*^2^ = 0.52), relative (*p* < 0.001, *ή*^2^ = 0.74) and absolute W_max_ (*p* < 0.001, *ή*^2^ = 0.75), SBP (*p* < 0.001, *ή*^2^ = 0.43), DBP (*p* < 0.001, *ή*^2^ = 0.41) and MAB (*p* < 0.001, *ή*^2^ = 0.50). In both groups, post-hoc tests showed significant increases in VO_2max_ and W_max_ and decreases in BP values (Figure 3). There were no significant between-group differences in the changes of any CRF or BP measure. Group-specific pre- and post-intervention values of all CRF and BP outcomes are shown in Table 4. 

### 3.5. Blood Markers

There was a significant main effect of time on fasting glucose (*p* < 0.001, *ή*^2^ = 0.27), LDL (*p* = 0.006, *ή*^2^ = 0.19), ALT (*p* = 0.026, *ή*^2^ = 0.13) and GGT (*p* = 0.005, *ή*^2^ *=* 0.19). Post-hoc tests showed significant reductions in GGT in the PRO-HIIT group (−2 U/L, 95% CI: −3 to −1 U/L, *p* = 0.006). In the PLA-HIIT group, there were significant reductions in LDL (−8 mg/dL, 95% CI: −15 to −2 mg/dL, *p* = 0.015) and ALT (−3 U/L, 95% CI: −5 to −1 mg/dL, *p* = 0.014), and a trend for an increase in fasting glucose (+ 5 mg/dL, 95% CI: 2 to 8 mg/dL, *p* = 0.052). Group-specific values of all the assessed blood markers are shown in Table 5. 

### 3.6. Participants’ Supplement Evaluation 

Overall, only a few mild adverse events were reported after the consumption of the protein supplement and included: stomach pain (N = 1), bloating (N = 1), mild nausea (N = 2) and burping (N = 1). No complaints were reported in the PLA-HIIT group. On a 7-point rating scale (1 = not enjoyable at all, and 7 = extremely enjoyable) the protein supplement and the placebo were rated with average scores of 4.0 ± 1.5 and 5.0 ± 1.4, respectively. In the PRO-HIIT group, 63% of participants reported that they would consider continuing to use the supplement (70% in the PLA-HIIT group) after the termination of the study.

## 4. Discussion

To the best of our knowledge, the present study was the first to investigate the influence of a targeted post-session protein supplementation on physiological adaptations to LOW-HIIT in a cohort of previously sedentary individuals. The main findings were as follows: (i) As expected, eight weeks of LOW-HIIT improved VO_2max_ and some cardiometabolic markers, including BP and liver enzymes, but (ii) contrary to our hypothesis, the post-session supplementation of 40 g whey protein did not promote more favorable training adaptations compared to the placebo ingestion. 

The average increase in VO_2max_ following LOW-HIIT (~3.2 mL/kg/min) was in accordance with the values observed in previous studies from our group, involving healthy sedentary individuals and obese metabolic syndrome patients (ranging from 3.0 to 7.1 mL/kg/min) [19,20,21,22,23]. Given that VO_2max_ is a key indicator of health [5,6,7], with each 1 mL/kg/min increase being linked to a reduction in CVD-related mortality by 9% [46], this finding can be considered highly clinically meaningful. Notably, since a lack of time is one of the most frequently reported obstacles to participating in regular exercise, it is an important confirming result of this study that a LOW-HIIT protocol requiring less than 30 min per session can effectively improve CRF within only a few weeks. A decrease in BP following LOW-HIIT has been a consistent finding in the literature [47], including research from our lab [20,21,22,23], and may be associated with exercise-induced improvements in vascular function [48]. The average reduction in systolic BP by ~8 mmHg that was observed after the eight-week LOW-HIIT period is very likely to provide a clinically meaningful benefit, since each 5 mmHg decrease in systolic BP has been suggested to lower the risk of developing major cardiovascular events by ~10%, even at normal or high–normal baseline BP values [49]. In this context, we note that the average pre-intervention BP of our participants was in the normal range (i.e., ~127/81 mmHg), pointing to a powerful preventive anti-hypertensive effect of LOW-HIIT. Additionally, the significant impact of LOW-HIIT on some liver enzymes detected in the present study is in line with our previous findings showing improvements in ALT serum concentrations and the non-alcohol fatty liver disease (NAFLD) fibrosis score in obese metabolic syndrome patients, who performed 12 weeks of LOW-HIIT [23]. Furthermore, the significant reduction (PLA-HIIT group, Table 5) or trend towards a reduction (PRO-HIIT, *p* = 0.067) in serum LDL levels following LOW-HIIT is also in accordance with a previous meta-analysis reporting that HIIT-based exercise interventions can have beneficial effects on the lipid profile [50].

When it comes to improving exercise performance and maximizing adaptations to training, respectively, a large variety of nutritional supplements are frequently used by athletes and recreationally active individuals, including, for example, carbohydrates, protein, vitamins, minerals, and creatine. While protein is certainly one of the most popular supplements consumed by individuals involved in resistance training in order to promote gains in skeletal muscle mass [24,51], proper carbohydrate intake is undoubtedly the crucial nutritional strategy for maintaining blood glucose levels during prolonged endurance exercise and for replacing depleted muscle glycogen stores after exercise [24,52,53]. Although the role of protein supplementation in endurance-training adaptations has been comparatively little examined, it has been hypothesized that individuals performing endurance-type training programs may also benefit from additional protein, particularly when consumed after exercise [27]. These suggestions are mainly derived from a small number of studies, showing that post-endurance-exercise protein ingestion may increase myofibrillar synthesis and/or activators of mitochondrial biogenesis such as peroxisome proliferator-activated receptor gamma coactivator-1 alpha (PGC-1α) [54,55,56]. Given that mitochondrial function and capacity are closely related to CRF [57] and cardiometabolic health [58,59], the potential beneficial impact of post-exercise protein ingestion on maximizing the training-related adaptations of the mitochondria, as detected in previous studies, is encouraging. However, it has also been pointed out that the evidence of the role of protein supplementation on endurance-training adaptations is still rather inconclusive. The current knowledge is limited by the lack of longer-term interventions (i.e., >6 weeks) and the fact that previous studies mainly focused on measuring muscle protein synthesis and biochemical endpoints (e.g., specific enzymes regulating mitochondrial biogenesis), which certainly contributed to a better understanding of the underlying physiological mechanisms but did not necessarily translate into proof of improved CRF and performance outcomes [27].

In contrast to our hypothesis, we did not detect any beneficial effect of a post-session protein supplementation on changes in CRF and cardiometabolic health measures following eight weeks of LOW-HIIT when compared to a placebo, which could be due to several factors. First, it is important to consider that previous studies that observed greater gains in VO_2max_ when training was combined with a post-session protein intake all used a substantially higher training volume (typically continuous, longer-duration aerobic exercise of 45–60 min duration/session) [54,60,61], while our LOW-HIIT protocol required as little as 14–24 min/session. Consequently, the total exercise volume of LOW-HIIT may simply not have been enough to cause an increased need for additional protein, and thus limiting the impact of the post-session supplementation. Second, both groups in the present investigation consumed adequate amounts of protein in their daily diet, which could have masked the potentially beneficial effects of protein supplementation. Third, it has been reported that repeated ingestions of 20 g protein during the post-exercise recovery period appear to be more effective compared to a single administration, at least when it comes to stimulating muscle protein synthesis after a resistance training session [62]. Accordingly, in two previous studies that observed greater VO_2max_ improvements when endurance training was combined with protein supplementation, the supplements were ingested repeatedly. More specifically, a first dose was administered immediately after the termination of exercise and another either 1 h later [55] or before sleep [60], respectively. Thus, it might be speculated that in the present study, a greater effect could have been achieved with a different protein-feeding pattern (i.e., repeated ingestions during the post-exercise period instead of a single administration). However, for compliance and monitoring reasons, we decided to administer only a single post-session protein dose, which may also be more representative of the supplementation habits among most individuals engaged in exercise routines in the real world. Moreover, it has previously been demonstrated that a single ingestion of 40 g whey protein following whole-body resistance exercise resulted in a significant (and, compared to 20 g, greater) stimulation of myofibrillar protein synthesis in healthy individuals [44]. Fourth, the self-reported daily carbohydrate intake among study participants was in the lower range of the recommendations for individuals involved in an exercise program [24], which could have mitigated improvements in the study outcomes. However, daily carbohydrate intake was similar between PRO-HIIT and PLA-HIIT and would therefore affect both groups in a similar manner. Fifth, previous research indicated that the improvement in VO_2max_ after a period of six weeks of endurance training was mainly attributed to an increase in cardiac output and blood oxygen-transport capacity, while skeletal muscle adaptations (i.e., capillarization, mitochondrial volume density) contributed less to VO_2max_ adaptations [63]. Thus, it is conceivable that an exercise intervention over a longer period would have been necessary to reveal more benefits of protein supplementation to changes in physiological variables involved in CRF and cardiometabolic measures. Taken together, our findings suggest that a single ingestion of 40 g whey protein immediately ingested after each session does not seem to augment adaptations of CRF and cardiometabolic measures in response to eight weeks of LOW-HIIT in previously sedentary healthy individuals.

There are some limitations that should be considered when interpreting the results of this study. First, we note that, except for the three days each at study entry and during the last intervention week, we did not control participants’ normal nutritional intakes and daily physical activities beyond the LOW-HIIT sessions. Moreover, we did not strictly standardize participants’ diet throughout the study period, with the exception of providing them nutritional recommendations at study entry in the form of handouts and recipes. Thus, although there were neither significant differences in daily nutrition, daily physical activity or MET-hours between the two recording periods nor between both groups, we cannot rule out that potential within- or between-group variations in participants’ nutrition, habitual physical activities or physical demands at their jobs during the non-recorded days of the eight-week intervention period could have affected the adaptations to LOW-HIIT. However, we highlight that it was the aim of our investigation to explore whether a targeted post-session protein supplementation could maximize the effects of LOW-HIIT without fundamentally influencing our participants’ habitual daily nutrition. Second, some of our conclusions are based on self-reported food- and physical-activity-recording data. In this regard, it must be kept in mind that individuals rather tend to underestimate their food intakes and to overestimate their physical activity levels during recording periods and/or that the recording per se may (unconsciously) affect the eating and physical activity behavior [64], although we suppose that the thorough explanation and briefing on how to record food intake and physical activity should have decreased the degree of potential errors. Third, we administered a fixed dose of 40 g protein after each LOW-HIIT session, and it could be argued that it would have been a better approach to match the supplement dosing to each participant’s body weight. We note, however, that it is not unusual that post-exercise protein dose recommendations are expressed as absolute values [24] and other previous studies applying a supplementation strategy with fixed amounts observed significant effects on VO_2max_ [60,61] or myofibrillar muscle protein synthesis [44,54], for example. Fourth, we acknowledge that we focused on evaluating the effects of post-session protein ingestion on CRF and cardiometabolic measures, but we did not investigate any specific markers associated with mitochondrial adaptation or muscle capillarization. Thus, we cannot rule out that we overlooked some relevant differences in skeletal muscle adaptations between PRO-HIIT and PLA-HIIT. Finally, we note that three participating women (PRO-HIIT, N = 1; PLA-HIIT, N = 2) were using contraceptive pills, which could potentially have an influence on blood pressure [65]. However, due to the very small number of women using contraceptive pills and the comparable distribution between both groups, we did not expect a meaningful impact on our results. Moreover, we highlight that the pre- and post-intervention examinations were standardized according to the individual menstrual-cycle phase of all female participants.

In spite of these limitations, this is, to our knowledge, the first double-blind randomized placebo-controlled study to investigate the effects of a targeted post-LOW-HIIT-session protein supplementation on longer-term adaptations of CRF and cardiometabolic outcomes in previously sedentary individuals.

## 5. Conclusions

Our data indicate that a post-session protein supplementation in the form of a single dose of 40 g whey protein does not enhance the adaptative response of CRF and cardiometabolic markers to eight weeks of LOW-HIIT in healthy, previously sedentary individuals compared to a placebo. Thus, the practical take-home message from this study is that individuals who consume adequate amounts of protein in their daily diet (i.e., ≥1.0 g/kg) do not seem to specifically benefit from ingesting a protein supplement after a session of LOW-HIIT.

## Figures and Tables

**Table 1:** Caloric value and macronutrient composition of supplements (per 100 g).

Variable	Protein ^1^	Placebo ^2^
Caloric value (kcal)	360	384
Protein (g)	87	0
Carbohydrates (g)	≤1	96
Fat (g)	1	0

^1^ Whey protein, ^2^ maltodextrin.

**Table 2:** Nutritional intakes and physical activity pre-intervention and during the last week of intervention.

Variable	PRO-HIIT (N = 19)		PLA-HIIT (N = 20)	
	T-1	T-2	T-1	T-2
Nutrition				
Energy (kcal/day)	2175 ± 608	2158 ± 787	2063 ± 633	2021 ± 594
Protein (g/day)	91 ± 42	84 ± 40	86 ± 35	77 ± 23
Protein (g/kg/day)	1.2 ± 0.5	1.1 ± 0.5	1.1 ± 0.6	1.0 ± 0.4
Fat (g/day)	80 ± 31	80 ± 31	86 ± 32	74 ± 25
Fat (g/kg/day)	1.1 ± 0.4	1.1 ± 0.4	1.1 ± 0.4	1.0 ± 0.4
Carbohydrates (g/day)	238 ± 69	240 ± 99	208 ± 80	226 ± 86
Carbohydrates (g/kg/day)	3.2 ± 1.0	3.2 ± 1.2	2.7 ± 1.2	3.0 ± 1.2
Fibers (g/day)	24 ± 10	25 ± 12	21 ± 8	22 ± 10
Physical activity ^1^				
Light PA (hrs/week)	2.6 ± 1.7	3.6 ± 2.5	3.8 ± 2.0	4.3 ± 2.1
Moderate PA (hrs/week)	0.7 ± 0.7	0.7 ± 0.9	0.5 ± 0.6	0.6 ± 0.6
MET hours/week	8.2 ± 3.4	9.3 ± 4.8	8.4 ± 5.3	9.9 ± 4.1

Values are given as mean ± SD. T-1 = pre-intervention, T-2 = last of week of intervention, PA = physical activity. ^1^ Excluding LOW-HIIT.

**Table 3:** Body composition data pre- and post-intervention.

Variable	PRO-HIIT (N = 19)		PLA-HIIT (N = 20)	
	T-1	T-2	T-1	T-2
Weight (kg)	76.6 ± 16.6	76.4 ± 16.4	77.3 ± 14.8	77.5 ± 14.8
BMI (kg/m^2^)	24.4 ± 3.2	24.4 ± 3.1	24.9 ± 3.8	24.9 ± 3.8
FM (kg)	20.3 ± 5.4	19.9 ± 6.2	23.2 ± 8.4	23.5 ± 8.8
FM (%)	26.9 ± 5.0	26.2 ± 6.0	29.7 ± 8.1	30.1 ± 8.2
SMM (kg)	27.0 ± 7.4	27.0 ± 7.3	26.1 ± 6.3	26.7 ± 5.8
TBW (L)	41.2 ± 9.7	41.4 ± 9.6	39.9 ± 7.9	39.3 ± 8.2
Waist (cm)	82 ± 11	81 ± 12	79 ± 20	78 ± 20

Values are given as mean ± SD. T-1 = pre-intervention, T-2 = post-intervention, BMI = body mass index, FM = fat mass, SMM = skeletal muscle mass, TBW = total body water.

**Table 4:** Cardiorespiratory fitness and blood-pressure measures pre- and post-intervention.

Variable	PRO-HIIT (N = 19)		PLA-HIIT (N = 20)	
	Pre	Post	Pre	Post
VO_2max_ (mL/kg/min)	39.4 ± 5.4	42.2 ± 6.3 ^b^	34.9 ± 7.1	38.4 ± 7.3 ^c^
VO_2max_ (L)	3.0 ± 0.8	3.2 ± 0.9 ^b^	2.7 ± 0.7	3.0 ± 0.7 ^c^
W_max_ (W/kg)	3.1 ± 0.4	3.6 ± 0.5 ^c^	2.8 ± 0.5	3.2 ± 0.6 ^c^
W_max_ (W)	241 ± 64	271 ± 74 ^c^	216 ± 50	245 ± 55 ^c^
W_VT_ (W)	72 ± 8	79 ± 16	73 ± 13	77 ± 22
SBP (mmHg)	126 ± 15	119 ± 11 ^b^	128 ± 13	120 ± 10 ^c^
DBP (mmHg)	79 ± 8	76 ± 7 ^a^	83 ± 8	78 ± 7 ^c^
MAB (mmHg)	94 ± 10	90 ± 8 ^b^	98 ± 9	92 ± 7 ^c^

Values are given as mean ± SD. VO_2max_ = maximal oxygen uptake, W_max_ = maximal power output, W_VT_ = power output at ventilatory threshold, SBP = systolic blood pressure, DBP = diastolic blood pressure, MAB = mean arterial blood pressure. ^a^ (*p* < 0.05), ^b^ (*p* < 0.01), ^c^ (*p* < 0.001): significantly different from pre-intervention.

**Table 5:** Blood markers pre- and post-intervention.

Variable	PRO-HIIT (N = 19)		PLA-HIIT (N = 20)	
	Pre	Post	Pre	Post
Glucose (mg/dL)	91 ± 6	94 ± 8 ^b^	92 ± 9	97 ± 8
HbA_1c_ (%)	5.2 ± 0.2	5.2 ± 0.2	5.1 ± 0.2	5.1 ± 0.2
Triglycerides (mg/dL)	105 ± 90	98 ± 48	101 ± 38	95 ± 42
Cholesterol (mg/dL)	214 ± 65	212 ± 46	213 ± 43	199 ± 43
HDL (mg/dL)	63 ± 13	63 ± 13	58 ± 9	57 ± 10
LDL (mg/dL)	138 ± 44	132 ± 39	138 ± 36	130 ± 30 ^a^
ALT [U/L)	25 ± 15	22 ± 13	22 ± 12	19 ± 9 ^a^
AST (U/L)	30 ± 14	27 ± 13	24 ± 5	23 ± 4
GGT (U/L)	18 ± 9	16 ± 9 ^b^	21 ± 11	19 ± 9
hsCRP (mg/dL)	1.7 ± 2.5	1.7 ± 2.5	1.5 ± 1.8	1.4 ± 1.9

Values are given as mean ± SD. HDL = high-density lipoprotein cholesterol, LDL = low-density lipoprotein cholesterol, ALT = alanine aminotransferase, AST = aspartate aminotransferase, GGT = gamma glutamyl transpeptidase, hsCRP = high-sensitivity C-reactive protein. ^a^ (*p* < 0.05), ^b^ (*p* < 0.01): significantly different from pre-intervention.

## Data Availability

The datasets generated and analyzed during the current study are not publicly available but are available from the corresponding author on reasonable request.
